# Correlation between [^68^Ga]Ga-FAPI-46 PET Imaging and HIF-1α Immunohistochemical Analysis in Cervical Cancer: Proof-of-Concept

**DOI:** 10.3390/cancers15153953

**Published:** 2023-08-03

**Authors:** Kgomotso M. G. Mokoala, Ismaheel O. Lawal, Letjie C. Maserumule, Meshack Bida, Alex Maes, Honest Ndlovu, Janet Reed, Johncy Mahapane, Cindy Davis, Christophe Van de Wiele, Gbenga Popoola, Frederik L. Giesel, Mariza Vorster, Mike M. Sathekge

**Affiliations:** 1 Department of Nuclear Medicine, University of Pretoria, Pretoria 0028, South Africa; kgomotso.mokoala@up.ac.za (K.M.G.M.); ismaheel.opeyemi.lawal@emory.edu (I.O.L.); letjie.maserumule@up.ac.za (L.C.M.); alex.maes@azgroeninge.be (A.M.); honest.ndlovu@sanumeri.co.za (H.N.); drjanreed@gmail.com (J.R.); sbahtherapy@gmail.com (C.D.); cvdwiele@hotmail.com (C.V.d.W.); 2 Nuclear Medicine Research Infrastructure (NuMeRI), Steve Biko Academic Hospital, Pretoria 0001, South Africa; 3 National Health Laboratory Services, Department of Anatomical Pathology, Pretoria 0001, South Africa; meshack.bida@nhls.ac.za; 4 Katholieke University Leuven, 3000 Kortrijk, Belgium; 5 Department of Radiography, University of Pretoria, Pretoria 0028, South Africa; johncy.mahapane@up.ac.za; 6 Department of Diagnostic Sciences, University Ghent, 9000 Ghent, Belgium; 7 Lincolnshire Partnership NHS Foundation Trust, St George’s, Lincoln, Lincolnshire LN1 1FS, UK; gbenga.popoola@nhs.net; 8 Department of Nuclear Medicine, Medical Faculty, University Hospital Dusseldorf, Heinrich-Heine-University, 40225 Düsseldorf, Germany; frederik.giesel@med.uni-duesseldorf.de; 9 Department of Nuclear Medicine, University of Kwazulu Natal, Durban 4001, South Africa; vorsterm1@ukzn.ac.za

**Keywords:** [^68^Ga]Ga-FAPI PET/CT, FAPI-TV, cervical cancer, HIF-1α, immunohistochemistry

## Abstract

**Simple Summary:**

Hypoxia is a phenomenon common in cervical cancer. Both the presence and function of CAFs are upregulated in a hypoxic environment. A key factor in the physiological response to hypoxia is hypoxia-inducible factor-1alpha (HIF-1α). We hypothesized that [^68^Ga]Ga-FAPI PET may be used as an indirect tracer for mapping hypoxia by correlating the image findings to pathological analysis of HIF-1α expression. The maximum and mean standardized uptake value (SUVmax and SUVmean) and FAPI tumor volume (FAPI-TV) were documented. There was uptake of tracer in the pelvis (cervix region) in all patients studied. All patients had lymph node metastases, while only six patients had distant visceral or skeletal metastases. The average FAPI-TV for patients with additional sites of metastases was higher than those without. Immunohistochemistry revealed varying intensities of HIF-1α expression in all tested samples. The presence of skeletal metastasis was correlated to the HIF-1⍺ staining (percentage distribution). Furthermore, the FAPI-TV was a better predictor of metastatic disease than the SUVmax.

**Abstract:**

Hypoxia leads to changes in tumor microenvironment (upregulated CAFs) with resultant aggressiveness. A key factor in the physiological response to hypoxia is hypoxia-inducible factor-1alpha (HIF-1α). [^68^Ga]Ga-FAPI PET imaging has been demonstrated in various cancer types. We hypothesized that [^68^Ga]Ga-FAPI PET may be used as an indirect tracer for mapping hypoxia by correlating the image findings to pathological analysis of HIF-1α expression. The [^68^Ga]Ga-FAPI PET/CT scans of women with cancer of the cervix were reviewed and the maximum and mean standardized uptake value (SUVmax and SUVmean) and FAPI tumor volume (FAPI-TV) were documented. Correlation analysis was performed between PET-derived parameters and immunohistochemical staining as well as between PET-derived parameters and the presence of metastasis. Ten women were included. All patients demonstrated tracer uptake in the primary site or region of the primary. All patients had lymph node metastases while only six patients had distant visceral or skeletal metastases. The mean SUVmax, SUVmean, and FAPI-TV was 18.89, 6.88, and 195.66 cm^3^, respectively. The average FAPI-TV for patients with additional sites of metastases was higher than those without. Immunohistochemistry revealed varying intensities of HIF-1α expression in all tested samples. There was a positive correlation between the presence of skeletal metastases and staining for HIF-1α (r=0.80;p=0.017). The presence of skeletal metastasis was correlated to the HIF-1⍺ staining (percentage distribution). Furthermore, the FAPI-TV was a better predictor of metastatic disease than the SUVmax.

## 1. Introduction

The tumor microenvironment consists of blood vessels, fibroblasts, immune cells, and a host of other cells [1]. Cancer-activated fibroblasts (CAFs) constitute the major component of the tumor stroma which in turn makes up 80% of the tumor mass [1]. The presence of these fibroblast cells is linked to increased aggressiveness, angiogenesis, and tumor progression, which subsequently leads to a poorer outcome [2]. The role of cancer-activated fibroblasts in cervical cancer progression has been investigated [3,4,5]. The presence of these cancer-activated fibroblasts was associated with a poorer prognosis [6].

Hypoxia is another component of the tumor microenvironment in solid tumors such as cervical cancer. Hypoxia-inducible factor-1α is an important molecular marker of hypoxia that regulates the activity of downstream genes and molecules that have varying effects on the tumor environment and behavior. The influence of hypoxia on fibroblast reprogramming and their tumor promoting functions has been documented [7,8]. The release of paracrine signaling molecules, such as transforming growth factor β (TGF β) and β fibroblast growth factor (β FGF) in the presence of hypoxia, leads to the reprogramming of progenitor cells into CAFs [9]. The work by Kugeratski et al. further proved that hypoxia aggravated the pro-angiogenic functions of CAFs [10]. This work has also been confirmed by other authors in other tumors [11].

Fibroblast activation protein (FAP) from the family of non-classical proteases, may also be termed seprase, propyl endopeptidase FAP, or antiplasmin-cleaving enzyme (APCE) [12]. It is rarely expressed in healthy human tissues except bone marrow stromal cells, pancreatic α cells, uterine stroma, some dermal fibroblasts, and in human placenta [12]. Cancer-activated fibroblasts express FAP and FAP is thus an important marker of CAFs. The FAP has been explored as a theragnostic molecule for diagnostic and therapeutic applications [13]. The use of FAP-specific inhibitors as [^68^Ga] Ga-FAPI which is a tumor-targeting radiopharmaceutical has opened up a new area of cancer research [12,14,15]. Early work on this tracer has demonstrated impressive uptake in various cancer types including cervical cancer [14].

The role of [^68^Ga]Ga-FAPI is still being explored as scores of papers are being published demonstrating the use of this tracer in various oncologic and non-oncologic applications [16]. It was found to have comparable performance to [^18^F]F-FDG PET in patients with head and neck carcinoma and soft tissue sarcomas [17,18], while it outperformed [^18^F]F-FDG PET in abdominal and pelvic malignancies (gastric, ovarian, hepatocellular, and bladder) [19,20,21,22,23]. It also fared well in IGG4 related diseases [24]. In gynecological malignancies, [^68^Ga]Ga-FAPI, displayed higher accuracy for detecting malignant lesions as is seen in ovarian cancer with carcinomatosis [19]. In a study that evaluated the use of [^68^Ga]Ga-FAPI in cervical cancer, the authors correlated their imaging findings with histopathological results from surgical specimen [25]. They found that [^68^Ga]Ga-FAPI showed better tumor-to-background ratios in both primary and metastatic diseases [25]. These studies demonstrate the complementary role of [^68^Ga]Ga-FAPI PET imaging in gynecological malignancies.

In view of the role of hypoxia in the upregulation of CAFs, we postulated that [^68^Ga]Ga-FAPI may serve as a surrogate marker of hypoxia. This study aimed to correlate [^68^Ga]Ga-FAPI PET imaging findings to immunohistochemical staining for HIF-1α.

## 2. Materials and Methods

### 2.1. Patients

We prospectively recruited patients with cervical cancer as part of an ongoing study. The study was approved by the Human Research Ethics Committee of the University of Pretoria (protocol number: 881/2019). Patients with locally advanced disease were referred for [^18^F]F-FDG PET/CT for initial staging prior to radiation therapy and others were referred for suspected disease recurrence. These patients were recruited to undergo an additional [^68^Ga]Ga-FAPI PET/CT scan. All patients provided an informed consent. All procedures were performed in accordance with the ethical standards of the institutional research committee in alignment with the 1964 Helsinki Declaration and its latter amendments.

### 2.2. [^68^Ga]Ga-FAPI PET/CT Procedure

The synthesis of [^68^Ga]Ga-FAPI-46 was performed as previously described [15]. Briefly, we obtained ^68^Ga from our in-house ^68^Ge/^68^Ga generator (iThemba LABS, Somerset West, South Africa). The FAPI peptide was obtained from SOFIE Biosciences, Inc., Culver City, CA, USA. The radiolabeling was conducted in-house and the labelling efficiency exceeded 95%. There was no specific patient preparation applied for the [^68^Ga] Ga-FAPI PET scan. The mean injected activity of [^68^Ga]Ga-FAPI was 5 ± 0.6 mCi/185 ± 22.2 MBq (range: 4.2–6 mCi). After an uptake period of 60 min, we acquired whole-body (vertex to mid-thigh) images. PET imaging was acquired in 3D mode at 3 min per bed position. Ordered subset expectation maximization (OSEM) iterative reconstruction algorithm (four iterations, eight subsets) was performed on the images followed by post-reconstruction filtering. The CT scan was a non-diagnostic scan and was performed in line with recommendations and as per our departmental protocol (previously published [26]). All patients were imaged on one of two systems, namely the Biograph 40 Truepoint PET/CT scanner (Siemens Medical Solutions, Hoffman Estates, IL, USA) and the Biograph Vision 450 PET/CT scanner (Siemens Medical Solutions, IL, USA).

### 2.3. Image Analysis

Semiquantitative analyses were used to quantify the [^68^Ga]Ga-FAPI uptake, and this includes maximum and mean standardized uptake values (SUVmax and SUVmean) and the FAPI-tumor volume (FAPI-TV) of the primary lesion. The FAPI-TV was computed in a manner identical to the [^18^F]F-FDG PET-derived metabolic tumor volume (MTV) analysis. The primary cervix lesion was identified, and a semi-automatic spherical volume of interest (VOI) was drawn around it on the transaxial images using a 3D isocontour of 41%. Qualitative analysis of the [^68^Ga]Ga-FAPI PET/CT images was performed by 2 board-certified nuclear medicine physicians with at least 10 years’ experience each in reporting PET/CT scans. Areas of increased tracer uptake not conforming to the known physiologic biodistribution of [^68^Ga]Ga-FAPI or benign/non-cervical lesions in the patients were considered malignant. Malignant lesions were described as local (due to primary local cervical cancer or local recurrence of cervical cancer) or metastatic. Metastatic lesions were categorized as nodal, skeletal, or visceral metastases. Any areas of disagreement were settled by consensus. Syngo software (Siemens Medical Solutions, Hoffman Estates, IL, USA) was used to reconstruct the images and both fused and unfused data were viewed in all planes.

### 2.4. Immunohistochemistry with HIF-1α

Endocervical biopsy specimens that were taken at the time of initial diagnosis were retrieved and used for the immunohistochemical analysis of HIF-1α expression. The slides were processed and analyzed by an experienced pathologist from the department of Anatomical Pathology, National Health Laboratory Services (NHLS). To map the expression of HIF-1α, we utilized HIF-1α monoclonal rabbit anti-human antibodies (clone EP118) (Abcam, Shanghai, China). Manual grading of the level of staining was obtained with a light microscope. We considered both the intensity of HIF-1α protein expression (nuclear) and the distribution in the tumor cells where the intensity was graded from 0 to 3 (negative to strong), and the distribution was assessed in percentages of 25% up to 100%. We considered any expression of HIF-1α for the analysis.

### 2.5. Statistical Analyses

Patient data including demographic and clinical data were analyzed using descriptive statistics. Frequencies, mean ± standard deviation (SD) and/or median (interquartile range, IQR) are used to present categorical and continuous variables, respectively. Statistical analysis was performed using SPSS, version 28.0 (IBM Corp, Amonk, New York, NY, USA). We used the KS test, Shapiro Wilks as well as Q-Q plots to assess the normalcy of the data. Given at least one variable proved not normally distributed and taking into consideration the small sample size, we decided to use less powerful non-parametric tests for data evaluation. The Spearman-rank test was used to assess the existence of a correlation between the various variables studied. The unpaired Mann–Whitney test was used to assess differences within variables for different subgroups. The Fisher-exact test was used to assess a difference in frequency in dichotomized disease stage (stage I + II versus III + IV) between HIV positive and HIV negative patients. Statistical significance was defined as *p*-values ≤ 0.05.

## 3. Results

### 3.1. Demographic Data

Ten women with cancer of the cervix uteri met the inclusion criteria and were included in the study. The mean age was 47 ± 12.76 years (range: 35–68). Four women were on antiretroviral therapy (ART) for human immunodeficiency virus (HIV) infection. The majority (8/10) of the patients were referred for a staging PET/CT and only 2 patients were referred for suspected recurrence following total abdominal hysterectomy with or without lymph node dissection. Squamous cell carcinoma was the most common histological subtype seen in eight of the 10 patients. A histological subtype of cervical cancer was adenocarcinoma in one patient and papillary serous carcinoma in another.

### 3.2. PET/CT Findings

All patients presented with tracer uptake in the primary site or region of the primary site (patients for restaging) as well as loco-regional and / or distant metastases. We found one or more sites of lymph node metastases in all patients included in the study. In addition to the lymph node metastases, three patients had visceral metastases while four had skeletal metastases (Table 1). The mean SUVmax, SUVmean, and FAPI-TV of the primary lesion or local recurrence was 18.89, 6.88, and 195.66 cm^3^, respectively. The average primary lesion or local recurrence FAPI-TV for patients with additional sites of metastases was higher than those without (222.98 cm^3^ vs. 154.32 cm^3^). All patients had an [^18^F]FDG PET/CT scan and the comparison of findings between the two studies can be found in Table 2 below.

### 3.3. Immunohistochemistry Findings

All but 2 endocervical biopsy specimen were retrieved for the immunohistochemical assessment for HIF-1α. There was positive staining of HIF-1α in all patients with varying degrees of intensity. The correlation between intensity and percentage distribution of HIF-1α and age as well as [^68^Ga]Ga-FAPI-46 PET derived parameters is depicted in Table 3.

### 3.4. Relationships between the Different Variables Studied

**Figure 1:**
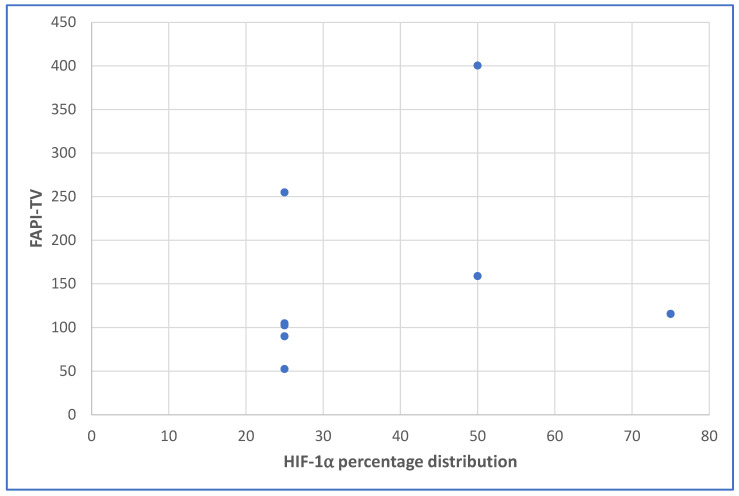
Scatterplot showing the relationship between the HIF-1alpha staining percentage distribution and the FAPI-TV.

**Figure 2:**
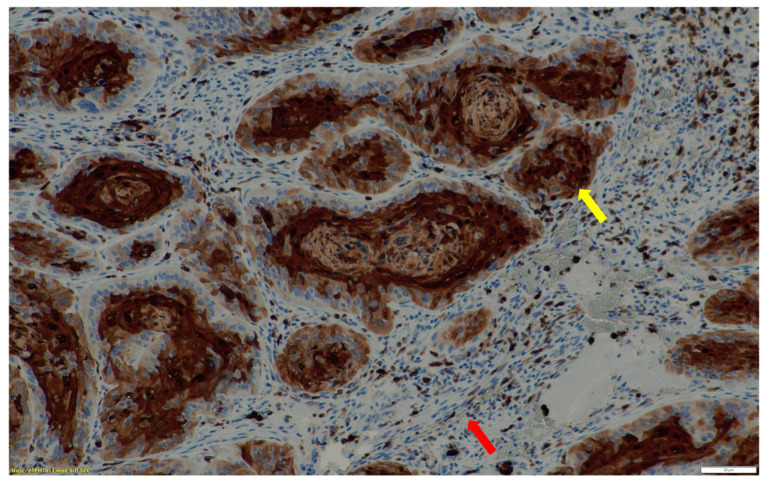
Immunohistochemical staining of HIF-1α expression in a moderately differentiated squamous cell carcinoma. Areas of low to no HIF-1α expression are indicated with a red arrow and those with HIF-1α expression are demonstrated with a yellow arrow. ×20 magnification.

**Figure 3:**
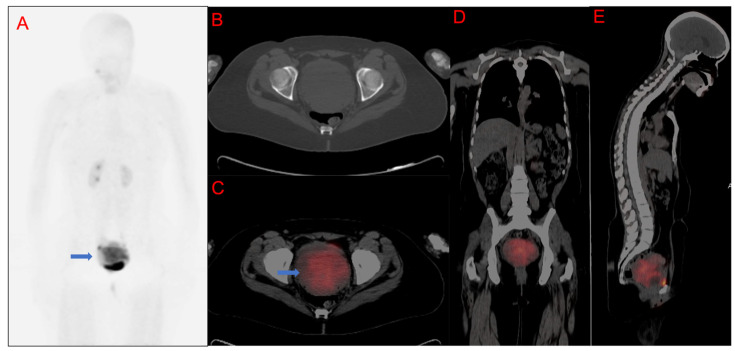
A 36-year-old female with moderately differentiated squamous cell carcinoma of the cervix. [^68^Ga]Ga-FAPI-46 maximum intensity projection images (MIP), (**A**). CT only axial slice through the pelvis, (**B**). Fused PET/CT axial images through the pelvis, (**C**). Whole-body fused PET/CT coronal images, (**D**). Whole-body fused PET/CT sagittal images, (**E**). Demonstrating a huge primary tumor (blue arrows) with intense [^68^Ga]Ga-FAPI-46 uptake. SUVmax = 24.41, FAPI-TV = 262.50 cm^3^.

**Figure 4:**
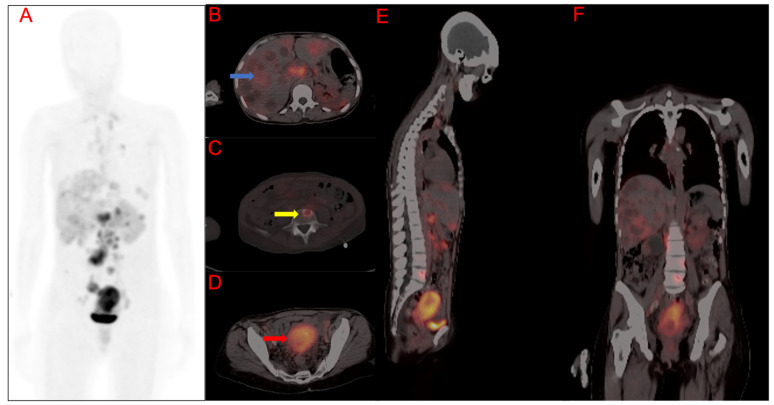
A 50-year-old female with poorly differentiated squamous cell carcinoma of the cervix. [^68^Ga]Ga-FAPI-46 PET/CT for staging demonstrated maximum intensity projected image (MIP) (**A**), fused PET/CT transaxial slice through the liver (**B**), fused PET/CT transaxial slice through the 4th lumbar vertebra (**C**), fused PET/CT transaxial slice through the pelvis (**D**), fused PET/CT whole-body sagittal (**E**), (blue arrow) and skeletal metastases (yellow arrow) and intense primary disease (red arrow) with uterine uptake, fused PET/CT whole-body coronal images demonstrate liver metastases (**F**). Primary cervix lesion SUVmax = 22.47, FAPI-TV = 115.54 cm^3^.

**Figure 5:**
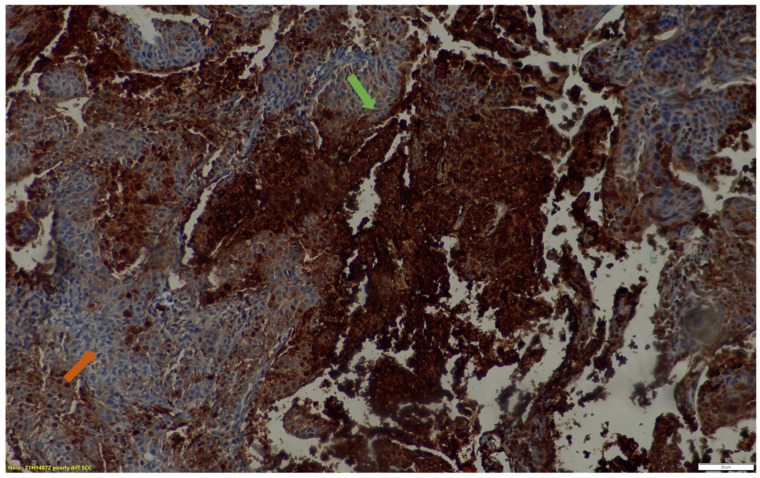
Immunohistochemical staining of the patient in Figure 3. above demonstrates HIF-1α expression in a poorly differentiated squamous cell carcinoma. Areas of intense HIF-1α expression are demonstrated with the green arrow while those with mild to moderate HIF-1α expression are indicated with an orange arrow. ×20 magnification.

The intensity of HIF-1α staining proved not significantly correlated to any of the variables studied, however there was a strong positive correlation with skeletal metastasis with a trend towards significance (r = 0.690, *p* = 0.058). Inversely, immunohistochemical staining distribution proved significantly higher in those patients that presented with bone metastases (*p* = 0.034) versus those who did not (median ± SD, 62 ± 18 versus 25 ± 10), and a trend towards higher FAPI-TV values (*p* = 0.087, Figure 1) was found in those patients that presented with bone metastases versus those who did not (median ±SD, 400 ± 128 cm^3^ versus 104 ± 83 cm^3^). Furthermore, FAPI-TV proved significantly smaller in HIV-positive patients when compared to HIV-negative patients (median ± SD, 96 ± 41 versus 258 ± 133, *p* = 0.033). Figure 2, Figure 3, Figure 4 and Figure 5 with immunohistochemical pictographs of HIF-1α staining and [^68^Ga]Ga-FAPI PET/CT images of two patients from our cohort.

### 3.5. Follow-Up

While we did not perform intensive survival analysis, we followed up with the patients to assess for the outcome. The duration of follow-up ranged from 83 weeks to 170 weeks. A total of 5 of the patients were treated palliatively with 5 sessions of 20 Gy external beam radiation (EBRT), while 3 of the patients received EBRT and brachytherapy (40 Gy × 16 sessions and 18 Gy × 2 sessions, respectively). We found that two out of the eight patients analyzed were still alive at the end of this analysis. Interestingly, the patients that are still alive had FAPI-TVs less than 100 cm^3^ (Table 4).

## 4. Discussion

All 8 biopsy specimen displayed HIF-1α expression of varying intensities implying the presence of hypoxia in all tumors albeit to different degrees. The distribution of HIF-1α expression was also variable ranging from 25% to 75% distribution within the tumor specimen. Hypoxia is a component of the tumor microenvironment in solid tumors and cervical cancer is no exception, and the presence of HIF-1α indirectly upregulates CAF’s and their pro-angiogenic functions in hypoxic states [27,28,29]. This very knowledge was the basis for our study which aimed to look at [^68^Ga]Ga-FAPI-46 PET imaging and correlate the findings (semi-quantitative parameters) to immunohistochemical HIF-1α expression and attempt to use [^68^Ga]Ga-FAPI as a surrogate marker of hypoxia.

We found a correlation between the presence of skeletal metastasis and the distribution of hypoxia (HIF-1α). Hypoxia and the presence of CAFs in the tumor environment have been linked to increased aggressiveness and progression of tumors [30,31,32]. Despite our modest study population, we were able to demonstrate a significant correlation between the distribution of the expressed hypoxia marker HIF-1α and metastasis, specifically skeletal metastases. Furthermore, we also found a higher FAPI-TV in patients with skeletal metastasis implying that the FAPI-TV of the primary lesion is a better indicator or predictor of metastases than the SUVmax or SUVmean. While the SUVmax has been used daily as a discriminator of malignant versus benign lesions on ^18^F-FDG PET imaging, it has its limitations and pitfalls [33,34,35]. Other semi-quantitative parameters such as the metabolic tumor volume (MTV) may be a better parameter to use and this has been found to be an independent prognostic factor in various malignancies, when using [^18^F]F-FDG PET/CT [36,37,38].

While HIV infection has been shown to increase the risk of malignant transformation of pre-cancerous cervix lesions to carcinoma in situ, there is a paucity of data regarding how this disease affects the clinical course of cervical cancer. In a systematic review by Ntekim et al., there was no difference in FIGO clinical stage at presentation between HIV-infected and HIV uninfected women [39]. We found a smaller FAPI-TV in HIV infected women than their HIV uninfected counterparts. In view of the adequate virological suppression, this finding suggests that HIV infection does not impact or influence the burden of disease in virally suppressed women. This contrasts with the work by Maiman and colleagues who reported on more advanced disease in HIV infected women [40]. The fact that all our patients were on highly active antiretroviral therapy (HAART) with undetectable viraemia may account for the above findings. In the era of HAART, women living with HIV with cervical cancer appear to have a similar clinical course to HIV uninfected women. This finding is consistent in most of our work where we found no differences in the tumor burden assessed on [^18^F]F-FDG PET/CT between HIV-infected and HIV uninfected women in various malignancies [41,42,43].

We found that [^68^Ga]Ga-FAPI-46 PET/CT performed well in women with cancer of the cervix uteri when compared to [^18^F]F-FDG PET/CT. It was able to detect all lesions in the primary site as well as distant metastatic sites. It performed equally well in the two patients referred for suspected recurrence, implying it may be used for staging and re-staging as is the case with [^18^F]F-FDG PET/CT. The work by Dendl and colleagues comparing [^68^Ga]Ga-FAPI-46 PET/CT and [^18^F]F-FDG PET/CT performance in various gynecological malignancies demonstrated the potential of this tracer for staging and follow-up of gynecological tumors including cervical cancer [44]. The mean SUVmax in cervical tumors was 15.22 which is similar to that of our cohort mean SUVmax 18.89. These high SUVmax values have also been seen in other tumor entities [14,45,46]. This higher level of tracer accumulation in the primary cervical cancer lesion allows for the delineation of the tumor from the diffusely increased background physiological activity in the normal uterus. Zhang et al. examined uterine uptake in patients who underwent a [^68^Ga]Ga-FAPI-04 PET for various indications [47]. They found that cervical cancer lesions concentrated [^68^Ga]Ga-FAPI-04 significantly more than the surrounding areas and that it may be performed in both staging and restaging [47]. Other authors also concluded that proper delineation of the primary tumor is still possible [25]. Despite this, it is still possible that the physiologic background tracer activity in the normal uterus may impair the accurate delineation of tumor extent on a [^68^Ga]Ga-FAPI PET/CT image. To surmount this, we describe the FAPI-TV in the tumor which set a isocontour threshold of 41%. By this threshold, we were able to compute the volume of the tumor expressing FAPI and excluding background activity in the normal uterus in a manner analogous to [^18^F]F-FDG PET-derived MTV.

In view of the fact that [^68^Ga]Ga-FAPI is a relatively new tracer, data are still being accumulated on the role it will play in oncologic imaging for routine patient management. Most clinical studies on [^68^Ga] Ga-FAPI have reported on SUVmax, SUVmean, and tumor-to-background ratios (TBR), however to the best of our knowledge, none have interrogated the use of what we termed the FAPI-TV. Also, our study reports the first assessment of [^68^Ga]Ga-FAPI PET imaging as a surrogate biomarker of tumor hypoxia using immunohistochemical staining for HIF-1α for correlation in a prospectively recruited study population. Despite the strengths of our study, there are important limitations that readers must be aware of. We described FAPI-TV extrapolated from MTV, an [^18^F]F-FDG PET-derived metabolic metrics. This extrapolation is similar to the practice where similar metric has been extrapolated from PET imaging with other tracers such as [^68^Ga]Ga-DOTA-peptides and ^68^Ga-prostate-specific membrane antigen [48,49]. Needless to say we have a modest sample size, therefore we cannot draw strong conclusions, however we can state that the trends we noticed need to be confirmed in larger studies.

## 5. Conclusions

The use of [^68^Ga] Ga-FAPI PET/CT in various malignancies including gynecological malignancies has been successfully established. Cervical cancer lesions and additional tumor sites were identified on [^68^Ga] Ga-FAPI-46 PET/CT imaging. We found a correlation between the immunohistochemical staining distribution of HIF-1α and the presence of skeletal metastases. Furthermore, we also found higher primary lesion FAPI-TVs in patients with skeletal metastases, implying that this parameter may outperform the SUVmax and may be a superior predictor of metastasis.

## Figures and Tables

**Table 1:** Demographic characteristics of the patients and [^68^Ga] Ga-FAPI PET/CT findings.

Patient No.	Age (Years)	Histological Subtype	FAPI-TV	Immunohistochemistry Staining Intensity of HIF-1alpha	Immunohistochemistry Staining Percentage Distribution	Metastases
1	63	Papillary serous ca	414.66	N/A		LN & Bone
2	62	SCC	102.47	1	25%	LN, lung & liver
3	35	SCC	89.95	2	25%	LN
4	42	SCC	158.90	2	50%	LN & Bone
5	38	SCC	254.92	1	25%	LN
6	39	SCC	52.39	1	25%	LN
7	37	SCC	400.35	2	50%	LN & Bone
8	36	SCC	262.50	N/A		LN
9	50	SCC	115.54	3	75%	LN, liver, spleen & bone
10	68	Adenocarcinoma	104.88	1	25%	LN & liver

Ca: carcinoma; SCC: squamous cell carcinoma; NA: not available; LN: lymph nodes.

**Table 2:** A side by side comparison of the PET-derived parameters from the [^18^F]F-FDG PET and the [^68^Ga]Ga-FAPI PET scans for the patients included in the analysis.

	[^18^F]F-FDG PET/CT			[^68^Ga]Ga-FAPI PET/CT		
Patient No.	SUVmax	SUVmean	MTV	SUVmax	SUVmean	FAPI-TV
2	13.16	5.39	110.23	10.46	5.29	102.47
3	19.47	6.6	111.51	29.02	4.79	89.95
4	14.4	6.21	158.91	15.64	6.29	158.90
5	16.41	5.89	204.32	19.32	5.54	254.92
6	18.21	7.52	88.13	29.21	9.93	52.39
7	19.43	8.54	359.09	23.48	7.83	400.35
9	25.09	6.6	140.75	22.79	5.93	115.53
10	16.95	6.28	111.93	22.47	5.25	104.88

SUVmax: maximum standardized uptake value, SUVmean: mean standardized uptake value, MTV: metabolic tumor volume, FAPI-TV: FAPI tumor volume.

**Table 3:** Correlation between age, metastases and PET-derived quantitative metrics with intensity and percentage distribution of HIF-1α on staining.

	Intensity of HIF-1α on Staining		Percentage Distribution of HIF-1α Staining	
Variable	r	*p* Value	r	*p* Value
Age	−0.287	0.491	0.014	0.974
Primary SUVmax	−0.300	0.470	0.027	0.948
Primary SUVmean	−0.117	0.782	0.220	0.601
Primary FAPI-TV	0.300	0.470	0.550	0.158
Visceral metastasis	−0.062	0.885	0.065	0.878
Skeletal metastasis	0.690	0.058	0.800	0.017

r: Spearman correlation coefficient. SUVmax: maximum standardized uptake value; SUVmean: mean standardized uptake value; FAPI TV: FAPI tumor volume.

**Table 4:** Analysis of imaging and immunohistochemistry findings in relation to the status of patient at follow-up.

Patient No.	FAPI-TV	Immunohistochemistry Staining Intensity of HIF-1α	Immunohistochemistry Percentage Distribution	Metastases	Alive (Yes/No)
*2*	102.47	1	25%	LN, Lung & liver	No
*3*	89.95	2	25%	LN	Yes
*4*	158.90	2	50%	LN & Bone	No
*5*	254.92	1	25%	LN	No
*6*	52.39	1	25%	LN	Yes
*7*	400.35	2	50%	LN & Bone	No
*9*	115.54	3	75%	LN, Liver, Spleen & Bone	No
*10*	104.88	1	25%	LN & Liver	No

## Data Availability

The data presented in this study are available on request from the corresponding author. The data are not publicly available due to limited space for the manucrsipt.
